# High Throughput Sequencing and Proteomics to Identify Immunogenic Proteins of a New Pathogen: The Dirty Genome Approach

**DOI:** 10.1371/journal.pone.0008423

**Published:** 2009-12-23

**Authors:** Gilbert Greub, Carole Kebbi-Beghdadi, Claire Bertelli, François Collyn, Beat M. Riederer, Camille Yersin, Antony Croxatto, Didier Raoult

**Affiliations:** 1 Center for Research on Intracellular Bacteria (CRIB), Institute of Microbiology, University Hospital Center, University of Lausanne, Lausanne, Switzerland; 2 Department of Cellular Biology and Morphology, University of Lausanne, Lausanne, Switzerland; 3 Proteomics Unit, Department of Psychiatric Neurosciences, Cery, Prilly-Lausanne, Switzerland; 4 Unité des Rickettsies, Faculté de Médecine, Université de la Méditerranée, Marseille, France; Duke University, United States of America

## Abstract

**Background:**

With the availability of new generation sequencing technologies, bacterial genome projects have undergone a major boost. Still, chromosome completion needs a costly and time-consuming gap closure, especially when containing highly repetitive elements. However, incomplete genome data may be sufficiently informative to derive the pursued information. For emerging pathogens, i.e. newly identified pathogens, lack of release of genome data during gap closure stage is clearly medically counterproductive.

**Methods/Principal Findings:**

We thus investigated the feasibility of a dirty genome approach, i.e. the release of unfinished genome sequences to develop serological diagnostic tools. We showed that almost the whole genome sequence of the emerging pathogen *Parachlamydia acanthamoebae* was retrieved even with relatively short reads from Genome Sequencer 20 and Solexa. The bacterial proteome was analyzed to select immunogenic proteins, which were then expressed and used to elaborate the first steps of an ELISA.

**Conclusions/Significance:**

This work constitutes the proof of principle for a dirty genome approach, i.e. the use of unfinished genome sequences of pathogenic bacteria, coupled with proteomics to rapidly identify new immunogenic proteins useful to develop in the future specific diagnostic tests such as ELISA, immunohistochemistry and direct antigen detection. Although applied here to an emerging pathogen, this combined dirty genome sequencing/proteomic approach may be used for any pathogen for which better diagnostics are needed. These genome sequences may also be very useful to develop DNA based diagnostic tests. All these diagnostic tools will allow further evaluations of the pathogenic potential of this obligate intracellular bacterium.

## Introduction

The recent availability of new generation sequencing technologies [1], [2] has provided unprecedented sequencing capacity, enabling the acquisition of genome-scale sequences at an extraordinary fast rate. These innovative techniques provide amazing opportunities for high-throughput structural and functional genomic researches and have been applied to date to a variety of contexts such as whole-genome sequencing [3] and resequencing [4], targeted resequencing [5], non coding RNA [6] or DNA-binding of modified histones [7], [8]. These high-throughput sequencing methods avoid the need for *in vivo* cloning and achieve a high accuracy. Even homopolymer problems, i.e. the major drawback of 454 pyrosequencing, may be overcome by reaching high sequence coverage [1]. These new technologies greatly reduce the work, time and expenses of such projects.

However, the relative short read length makes genome assembly problematic and their use in bacterial genomics has been fairly restricted to new strains closely related to already sequenced organisms to identify for example virulence factors [9], antibiotic resistance genes [10], or epidemiological markers [11]. Although improved techniques can now achieve paired-read information and longer reads [12], genomes still need a costly and time-consuming gap closure step, especially when containing highly repetitive elements such as transposases and recombination hot spots.

Still, complete genomic information is not necessarily needed and incomplete genome data obtained using high-throughput sequencing methods may potentially be informative enough to derive the pursued information. Moreover, the low time to results of such approaches (about 15 weeks [9]) is especially useful when genomic information are readily needed for instance in case of outbreaks (i) to search for the presence of specific pathogenicity island or virulence genes, (ii) to identify specific or multicopy gene targets in order to rapidly develop a reliable molecular diagnosis test, and (iii) to identify immunogenic proteins to set up a diagnostic tool for sero-epidemiological investigations or to develop a vaccine.

This strategy is particularly interesting for obligate intracellular bacteria such as members of the *Chlamydiales* order that lack a genetic manipulation system and only replicate within eukaryotic cells of different origins including humans, animals and amoebae [13]. One of them, *Parachlamydia acanthamoebae* strain Hall's coccus, was initially isolated from the water of an humidifier at the origin of a fever outbreak [14], and since then some evidences have accumulated suggesting the role of this species as an emerging human respiratory pathogen [15]. An emerging pathogen refers here to an agent that has been recently identified as pathogenic. Indeed, several serological and molecular studies supported a role of *P. acanthamoebae* in patients with community-acquired and aspiration pneumonia [16], [17], [18]. *P. acanthamoebae* also appeared to possibly cause bronchiolitis in children [19]. Moreover, pneumonia has been reproduced in a murine model following intranasal and intratracheal inoculation with *P. acanthamoebae* [20], [21]. Finally, the ability of *Parachlamydia* to resist to human macrophages [22], [23] further supported its human pathogenicity. Besides, the role of *P. acanthamoebae* in bovine abortion has been clearly demonstrated since the bacteria was detected by PCR, immunohistochemistry and electron microscopy in the placenta of aborted bovines [24]. The pathogenic potential of *Parachlamydia* towards humans and animals still remains largely unexplored since this strict intracellular bacterium does not grow on media routinely used for the detection of pathogens. To date, there are only few strains of *Parachlamydia acanthamoebae* available worldwide. Moreover, little information is available about strains from cattle and other animals, since no *Parachlamydia* strains have been isolated from animal samples by cell culture. It is thus important to develop new diagnostic approaches for *P. acanthamoebae* to better understand its epidemiological and pathogenic potentials in various human and animal diseases.

In this work, we undertook a proof of principle project that investigated the feasibility of combining genomic and proteomic approaches to rapidly identify immunogenic proteins. We showed that, even with relatively short reads from Genome Sequencer 20 (GS20) and after homopolymer correction through Solexa, we can gather almost the whole genome sequence of an emerging pathogen, allowing to analyze the proteome and to elaborate the first steps of an ELISA test, thus enabling to further evaluate its pathogenic role.

## Results

### Genome Sequencing

The pyrosequencing of *P. acanthamoebae* genomic DNA by two runs of GS20 yielded 566'453 reads of an average length of 111 nucleotides. In order to correct eventual frameshifts due to homopolymer errors, genomic DNA was also sequenced with Solexa technology, which produced 1'655'941 short reads of 36 bp that could be assembled in 8616 contigs. The latters were assembled with GS20 reads in 95 contigs larger than 500 bp, with a N50 size of 101'998 bp. The coverage obtained with 454 reads was of 17x whereas that obtained with Solexa reads was of 12x. The 95 contigs represents approximately 97% of the total genome and as many as 99.99% of all the non-repeated regions, i.e. when excluding contigs exhibiting a sequence depth higher than 30x with 454. As indicated by the total length of the contigs, the complete genome of *P. acanthamoebae* Hall's coccus stands around 3 Mb and was predicted to contain 4798 open reading frames larger than 90 nucleotides. More than 91% of the large contigs were covered with Solexa. The 1037 differences between Solexa and 454 were manually inspected. As many as 405 differences could be attributed to the presence of homopolymers and were corrected according to Solexa whereas the remaining 632 differences were mainly due to inaccurate Solexa contig ends and were not corrected.

### Identification of Immunoreactive Proteins

**Figure 1:**
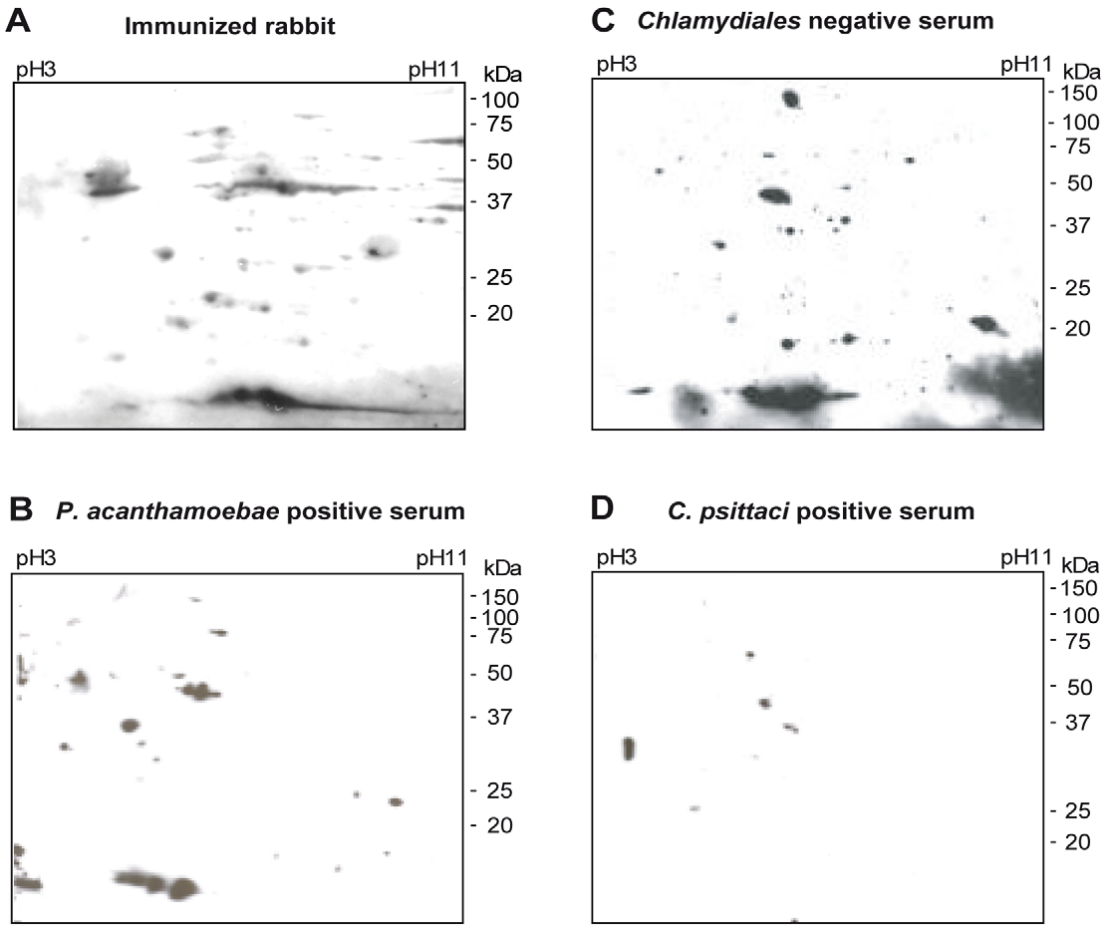
2D patterns of the immunoreactive proteins of *P. acanthamoebae*. Proteins of *P. acanthamoebae* separated by 2D gel electrophoresis were probed with (**A**) serum from immunized rabbit #1, (**B**) a *Chlamydiales* negative human serum, (**C**) a *P. acanthamoebae* positive human serum, and, (**D**) a *C. psittaci* positive human serum. Five immunogenic proteins are numbered in reference to the following figures.

**Figure 2:**
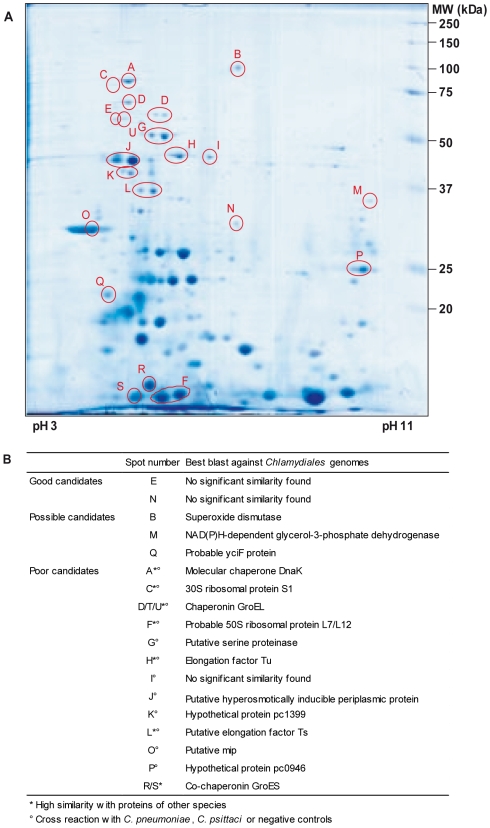
2D map and identification of *P. acanthamoebae* immunogenic proteins. **A**. Proteins reacting with at least one rabbit anti-*Parachlamydia* serum were excised from gel and analysed by MALDI TOF mass spectrometry. Spots successfully identified are numbered. Molecular mass standards are indicated on the right side of the gel. **B**. The potential of 18 immunogenic proteins for use in a serological diagnostic test was evaluated based on their reactivity with control sera and on their sequence similarity in BLASTP results (see **Table S2** for detailed analysis).

To identify immunoreactive proteins that could be used in a diagnostic test, total proteins of *P. acanthamoebae* elementary bodies were separated by 2D gel electrophoresis and either Coomassie blue-stained or transferred onto nitrocellulose membranes. Immunoblots were performed with sera of rabbits immunized with *P. acanthamoebae* and with human *P. acanthamoebae* positive sera (**Fig. 1A,C**). Spots corresponding to immunogenic proteins reacting with at least one rabbit anti-*Parachlamydia* serum were selected by computer-assisted matching of the Coomassie blue-stained gel and immunoblots, and further analyzed by mass spectrometry. Eighteen different proteins were identified (**Fig. 2A**), out of which 5 reacted only against sera from immunized rabbits and 13 reacted with both rabbit and human *Parachlamydia* positive sera. Some of these proteins, such as chaperonin GroEL (Hsp60), DnaK (Hsp70), elongation factor Tu and the ribosomal proteins S1 and L7/L12, were already known to be antigenic [25] (**Fig. 2B** and **Table S1**). Some classical *Chlamydiales* immunogenic proteins, such as 60 kDa cysteine-rich OMP, LcrE or CPAF protease [25], [26], [27] were not detected. Since the corresponding genes were found in our contig assembly, these proteins are likely poorly expressed in elementary bodies or when the bacteria are co-cultivated in amoebae. Membranes were also probed with control human sera, i.e. either completely negative for any member of the *Chlamydiales* order (**Fig. 1B**), or positive only for *C. psittaci* (**Fig. 1D**) or *C. pneumoniae* (see **Table S2**). Based on 2D immunoblots with control sera and blast analysis of the MS identified proteins, the best candidates for a diagnostic assay of *P. acanthamoebae* infection were determined (see **Table S2**). Antigens displaying a high sequence homology with similar proteins in other species as well as proteins cross-reacting with non specific or negative sera were discarded. The two best candidate proteins were selected for evaluation in an ELISA test (**Fig. 2B**).

### Western-Blot and ELISA of Proteins E and N

**Figure 3:**
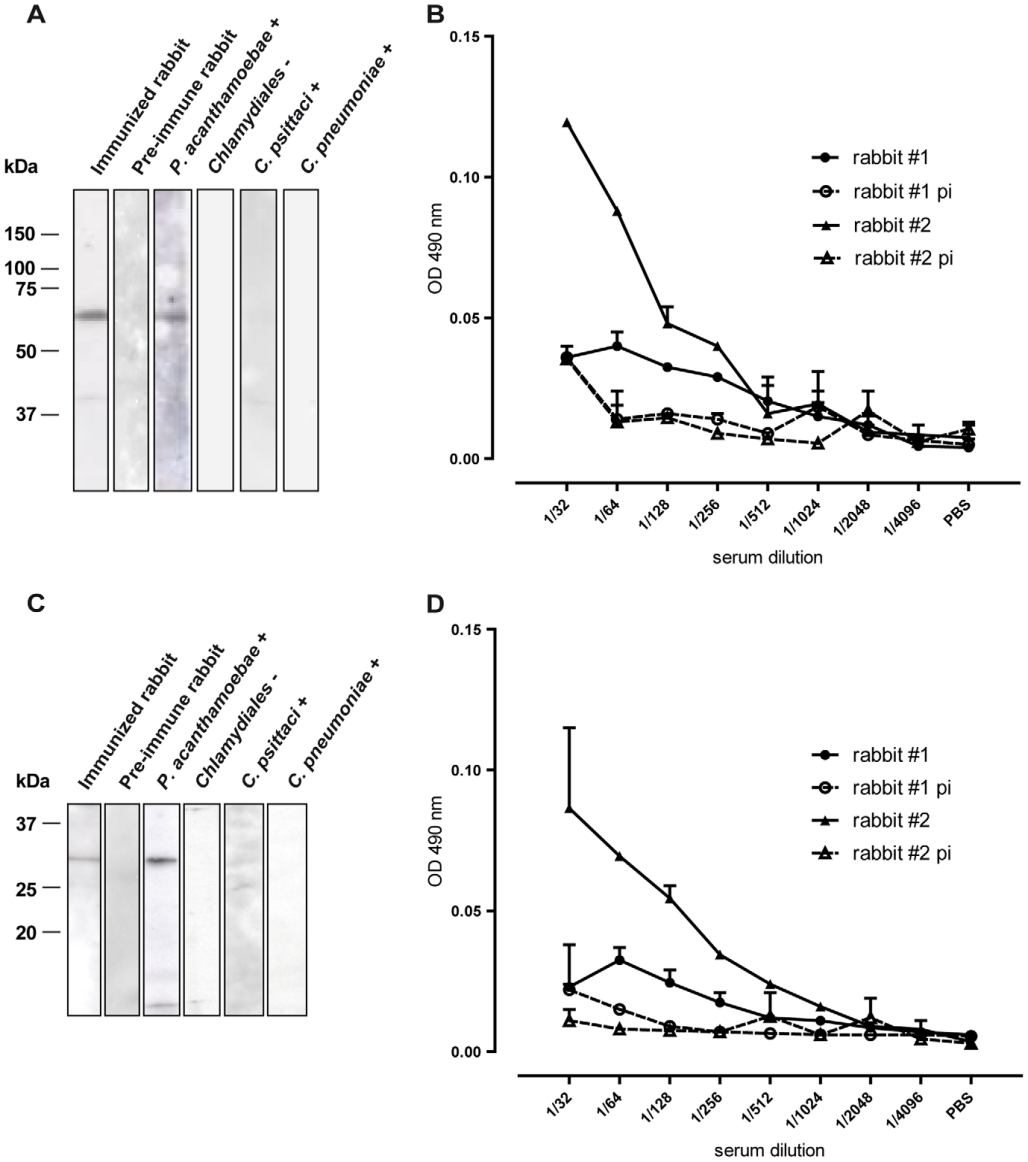
Western blot and ELISA with recombinant proteins E and N. Purified recombinant protein E (**A**) and N (**C**) were blotted on a nitrocellulose membrane and probed with a rabbit anti-*Parachlamydia* serum (rabbit #1), a rabbit pre-immune serum (rabbit #1), a *P. acanthamoebae* positive human serum, a *Chlamydiales* negative human serum, a *C. psittaci* positive human serum and a *C. pneumoniae* positive human serum. Proteins E (**B**) and N (**D**) were used as antigen in a direct ELISA. Sera from 2 rabbits immunized with *P. acanthamoebae* and pre-immune (pi) sera were tested in duplicates.

The parachlamydial protein E and N, which have no sequence homology with any known protein, were chosen to develop serological diagnostic tools. Recombinant proteins E (MW ∼58 kDA) and N (MW ∼30 kDA) were expressed in *E. coli* and purified thanks to a 6His tail fused to their N-terminal end. The purified recombinant proteins were detected by western blot with a rabbit anti-*Parachlamydia* serum or with a human *P. acanthamoebae* positive serum (**Fig. 3A,C**). Lower molecular weight bands also visible on these blots probably correspond to degradation products. Moreover, faint bands were detectable when protein E was probed with a *C. psittaci* positive serum indicating a low level of cross reaction with this organism. For both proteins, no signal was obtained when *Chlamydiales* negative or *C. pneumoniae* positive sera were tested.

When used as antigen in a direct ELISA assay, purified proteins E and N were detected by the sera of two rabbits immunized with *P. acanthamoebae* until a dilution value of 1/256 while only background reaction is observed with pre-immune sera at this and lower dilutions (**Fig. 3B,D**). For both proteins, a significant difference was observed in the level of reactivity of the two sera. However, both rabbit sera exhibited good antigen reactivity when tested by western-blot. Overall, these data demonstrate the potential of immunogenic proteins E and N for serological diagnostic tests that could be developed in the future.

### Comparison between Combined or Separated 454 and Solexa Approaches to Identify Proteins

In addition to identifying immunogenic proteins, the most abundantly expressed proteins of *P. acanthamoebae* elementary bodies were also analyzed. A total of 95 Coomassie blue-stained spots were analyzed by mass spectrometry and a reliable protein identification was obtained for 85 of them using the combined GS20 and Solexa sequences. Identification failed for 2 proteins due to the absence of signal by mass spectrometry and for 8 proteins due to the absence of hits in the genome-derived protein database. In many cases multiple spots on the gel corresponded to a single protein so that 61 different proteins were identified, including the immunoreactive proteins described above (see **Fig. S1**).

All 61 proteins identified using combined GS20 and Solexa sequences would also have been identified when using only the GS20 sequences-derived protein database. However, with uncorrected GS20 sequences, 4 ORFs presented a frameshift leading twice to a longer protein and twice to a premature end of the protein, i.e. splitting the ORF in two parts. Only 5 of the 61 proteins identified using combined GS20 and Solexa sequences were identical to the predicted ORFs using only Solexa sequences. The remaining 56 proteins were split between two to seven different small contigs preventing their accurate identification by Mascot. The limited performance of Solexa technology as compared to 454 is likely due to the short Solexa reads and the relative low sequence depth obtained.

### Most Abundantly Expressed Proteins and Virulence Genes

By BLAST against nr database, a function could be derived for half of the 61 proteins identified by mass spectrometry (for details, see **Table S1** and **Table S3**), whereas one fourth have homologs of unknown function in *Protochlamydia amoebophila* genome [33] or in other organisms. Finally, the remaining proteins exhibit no significant homology with any known amino acid sequence.

Our assembly also enabled the identification of several virulence genes present in *P. acanthamoebae* genome. In addition to the previously mentioned LcrE and CPAF protease, *P. acanthamoebae* encodes a complete type three secretion system (T3SS), including components of the secretory apparatus, translocators, T3SS specific chaperones and effectors [28]. Like in other *Chlamydiales* species, T3SS genes are spread in distinct conserved genomic clusters (see **Fig. S2**). Interestingly, *sctJ* and *sctC*, two genes encoding components of the secretory apparatus, are duplicated in the genome. Moreover, four clear homologs to *Protochlamydia amoebophila* nucleotide transporters (*ntt_1*, *ntt_2*, *ntt_3* and *ntt_4*) which play a key feature in energy parasitism [29], [30] have been identified in *P. acanthamoebae*. A fifth putative ADP/ATP translocase candidate was also detected but homologies were not sufficient to establish the substrate transport specificity. Finally, four genes belonging to the F-like conjugative DNA transfer operon located on the genomic island of *Protochlamydia amoebophila* [31] were also detected in *P. acanthamoebae* (*traU*, *traN*, *traF* and *pc1435*).

## Discussion

In case of outbreak due to a new pathogen, diagnostic tests must be developed rapidly. Genome sequence is an important resource to develop various tools for molecular and serological diagnostic, specific monoclonal antibodies production or vaccine design. With the availability of high throughput sequencing strategies such as the widely used GS20/GSFLX [1], large sequence datasets are now obtained within very short time. However, the costly and time-consuming follow up necessary to close the gaps delays the release and accessibility of most genome sequences. As shown for *Francisella tularensis*, a rapid comparative genome analysis can be successfully applied on unfinished contigs enabling to uncover genomic rearrangements or gene mutations that could be involved in an increased strain virulence and resistance [9]. A similar approach was also proposed to study the role of *Helicobacter pylori* in chronic gastric infection by analyzing genetic changes in this species over time or between infected humans [32].

A rapid and public availability of raw genome data from an emerging pathogen at the origin of an outbreak is critical to permit the development of various diagnostic tools by medical microbiologists. The delay before genome release is especially crucial in case of new pathogenic agents with the absence of available closely related genomes, i.e. absence of scaffold that may be used to facilitate assembly and gap closure steps. This problem was faced here for *P. acanthamoebae* with the availability of a single published genome within the *Parachlamydiacaeae* family, that of *Protochlamydia amoebophila* [33]. The presence of repeated elements in the genome significantly increases the number of contigs obtained, thus prolonging the gap closure. Although *Chlamydiaceae* genomes do not contain many transposases, the genome sequence of *Protochlamydia amoebophila* was much more invaded by such repeated components [33]. This suggests that sequence repetitions probably account for a large number of gaps in our own *Parachlamydia* genome project. Nevertheless, if these repeated elements can prevent an assembly in one unique contig, they do not hinder the availability of most coding sequences. Indeed, 90% of analyzed proteins could be identified, the remaining 10% being uncharacterized due either to the lack of mass spectrometry signal or to the lack of hits in the ORF database. Thus, although we could not determine the exact number of immunogenic proteins that have been missed due to the presence of the remaining gaps, we may estimate that only few (<10%) additional immunogenic proteins would have been identified if a complete genome sequence was available.

Our proteomic approach allowed us to detect 18 immunogenic proteins among which are several antigens already described as highly immunoreactive such as GroEL, DnaK or elongation factor Tu [25]. Five proteins represented good/possible candidates to develop a diagnostic test since exhibiting significant reactivity to sera taken from humans infected by *Parachlamydia* as well as from immunized rabbits and no cross-reactivity to sera from humans infected with *C. pneumoniae, C. psittaci* and negative controls. Then, we focused on only two of these five proteins, E and N, displaying no significant homology with any known amino acid sequence. Their potential to develop vaccine or diagnostic tools was suggested by western-blot and by preliminary ELISA tests despite the absence, in the heterologous protein expression system used, of post-translational modifications such as glycosylation or phosphorylation that might have resulted in poor serum recognition. Given its 96-well format, the ELISA test, once developed, would be very useful in large epidemiological studies to assess the precise seroprevalence of *Parachlamydia* antibodies in human population and to confirm the pathogenic role of this intracellular bacterium in human lower respiratory tract infections and in bovine abortion. Moreover, an ELISA based on a given immunogenic protein will be more specific than diagnostic microimmunofluorescence and western-blot assays based on whole bacterial proteins.

Interestingly, among the 85 ORFs identified using our dirty genome sequences, only 9 proteins could have been identified by Mascot versus protein sequences of the closest related bacteria available to date, *Protochlamydia amoebophila*, all of which are very conserved and, if immunogenic, would likely produce strong cross reactions when used in serological tests. In addition, among the five immunogenic proteins selected as good and possible candidates for the development of a diagnostic ELISA, none have been identified by Mascot versus SPTrembl database because of the differences between the peptides identified and the protein database. Moreover, the two proteins we considered as the best candidates have no homologs in other genomes and their sequences could not be derived from those of any related or unrelated bacteria.

This further emphasizes the need for a protein database directly derived from genome sequences of the studied emerging pathogen. Besides, rapid genome sequencing provides information useful not only for proteomics but also for comparative genomics, transcriptomics, cell biology and molecular biology. The availability of most genome sequences of a new emerging pathogen isolated during an outbreak may also be important to design molecular diagnostic tools, to define epidemiological marker as well as to identify virulence genetic traits and antibiotic resistance determinants.

**Figure 4:**
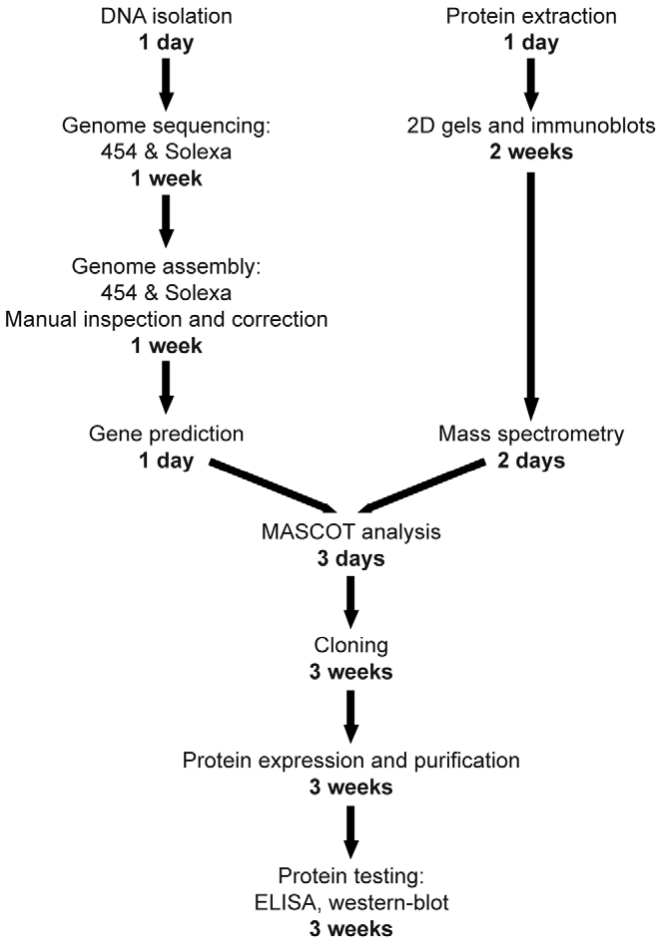
Time scale of a dirty genome approach combined with proteomics to develop serological diagnostic tools. Schematic representation of the main steps of genome sequencing, immunogenic proteins identification and testing of candidate proteins in an ELISA. In bold, approximate time necessary to complete each step.

The advantages of using mass spectrometry associated with an unfinished genome to identify immunogenic proteins, compared to other approaches such as phage display library [34], [35], comparative genomic [36] or systematic expression of all ORFs [37], resides mainly in the minimal necessary workload and in the rapidity of the method. Indeed, the whole process can take place in less than 4 months, with only 2 weeks necessary to obtain the contigs (**Fig. 4**). In addition, with the lowering of sequencing costs, the price of such an approach is highly competitive. Furthermore, constructing random expression libraries by fractionation of whole bacterial DNA would likely identify less immunogenic proteins since any plasmid carrying ORFs whose product is toxic will not be successfully expressed.

The 4 months that it takes to develop an ELISA may seem long as compared to the few weeks needed to develop a DNA-based test. However, detecting proteins represents a distinct advantage over detecting unique DNA sequences, and the availability of a serological test may especially prove very useful (i) to confirm positive PCR results (that may be false positive due to PCR contamination) and to better document a given case, (ii) to perform large seroepidemiological studies in order to precise the mode of transmission of a new pathogen and (iii) to investigate the possible role of a new bacterial pathogen in different clinical settings, such as pericarditis and endocarditis, for which valvular/pericardial fluid samples are not easily available. Similarly, when investigating patients with atypical pneumonia, serum samples are easier to obtain than lower respiratory tract specimen, especially when patients present a non-productive cough. Moreover, for several fastidious intracellular bacteria, even highly sensitive PCR tests may fail in detecting the agent at the infection site (i) due to relatively low bacterial load, e.g. sensitivity of only 50% to detect *Borrelia* in cerebrospinal fluid taken from patient with neurological Lyme disease [38], (ii) due to the presence of inhibiting molecules present in clinical samples, or (iii) due to “sampling bias” of PCRs tests performed on tissue samples, e.g. sensitivity of 60% of PCR on valve samples taken from patients with definite endocarditis [39].

Furthermore, the identification of immunogenic proteins also allows the development of species specific immunohistochemistry, that is useful (i) to confirm the presence of the pathogen in the tissue lesions, (ii) to analyze retrospectively various biopsy samples taken from patients with infection of unknown etiology and (iii) to shed some light on the underlying pathogenesis in vivo, by precising which cells are infected using double staining.

In summary, this work constitutes the proof of principle for a dirty genome approach, i.e. the use of unfinished genome sequences of pathogenic bacteria, coupled with proteomics to rapidly identify immunogenic proteins useful to develop a specific diagnostic test. Indeed, genomic information concerning new emerging pathogens must be placed at the scientific community's disposal as soon as possible, since their retention in order to close all gaps before genome publication is clearly medically counterproductive. This work demonstrated that 454/Solexa combined dirty genomes are sufficient and useful for medical downstream applications.

## Methods

### Ethics Statement

Human sera used in this work (see below) have been obtained from patients and control subjects, as part of prospective studies. These clinical studies have been accepted by the local ethical committee of the University of Lausanne. Both patients and controls gave their informed consent for various serological investigations including study of their serum reactivity against *Parachlamydia acanthamoebae*.

### Cultivation and Purification of *P. acanthamoebae*

*Parachlamydia acanthamoebae* strain Hall's coccus was grown in *Acanthamoeba castellanii* strain ATCC 30010 in peptone yeast-extract glucose broth (PYG) and purified using a sucrose barrier and a gastrographin gradient, as previously described [22].

### Chromosome Sequencing and Assembly

Purified *P. acanthamoebae* elementary bodies resuspended in PBS were lysed for DNA extraction with QIAamp DNA mini kit (Qiagen) according to the manufacturer protocol. Genomic DNA was pyrosequenced by two runs of Genome Sequencer 20 [1]. Genomic DNA was also sequenced using Solexa technology in Illumina Genome Analyzer [2]. Solexa sequences were assembled using Edena software [40] with parameter m = 16. Both GS20 runs only or GS20 runs and Solexa contigs were compiled in one assembly using Newbler software V1.1.02 with default parameters except for overlap size (45 nt) and identity score (95%). Differences between 454 and solexa contigs were manually inspected and corrected when necessary. In case of homopolymer discrepancy preference was generally given to Solexa when correcting frameshifts in protein coding region and in potentially non coding regions.

### Gene Prediction and Annotation

To improve the prediction of incomplete genes at contig ends, a stop encoding tag “CTAGCTAGCTAG” was added at both extremities of each contig. A reference proteome was created with all open reading frames (stop to stop ORF) for peptide identification. Besides, locally installed Glimmer v3.02 [41] trained on long ORFs of the concatenated contigs was then applied to predict gene position on contigs longer than 500 bp. All reported ORFs larger than 100 nt were submitted to BLASTP versus nr database and local InterProScan search. Finally, tRNAscan-SE [42] and RNAmmer [43] were used to find structural RNAs. Genes of special importance for this study were manually annotated. This Whole Genome Shotgun project has been deposited at DDBJ/EMBL/GenBank under the accession ACZE00000000. The version described in this paper is the first version, ACZE01000000.

### Crude Extract Sample Preparation and 2D Gel Electrophoresis

Bacterial cells resuspended in PBS were washed in 10 mM Tris, 5 mM MgAc, pH 8.0 and then lysed by 5 cycles of short-pulse sonication in lysis buffer (30 mM Tris, 7 M urea, 2 M Thiourea, 4% CHAPS, pH 8.5). Proteins were recovered by centrifugation at 6'000 *g* and their concentration determined using a Bradford assay (Quick Start™ Bradford Protein Assay, Biorad, Hercules, USA).

Two dimensional gel electrophoresis was performed as described by Centeno et al. [44] using approximately 150 µg (mini gels) or 600 µg (midi-gels) of total elementary bodies proteins. Proteins were visualized by Coomassie Blue staining or transferred to nitrocellulose for subsequent immunoblot analysis (see **Methods S1**).

### Human Sera

*P. acanthamoebae* positive human sera were described in previous studies where their positivity was assessed by immunofluorescence and western-blot [17], [45]. Control sera were taken from women with at term uneventful pregnancy [45]. *Chlamydiales* negative sera were tested negative by immunofluorescence for reactivity against various members of the *Chlamydiales* order (*P. acanthamoebae, W. chondrophila, S. negevensis, N. hartmannellae, C. trachomatis, C. pneumoniae* and *C. psittaci)*, *C. pneumoniae* positive sera were positive for *C. pneumoniae* but negative for all other *Chlamydiales* tested. *C. psittaci* positive human serum was taken from a patient who suffered from well-documented psittacosis [46].

### ELISA

Proteins E and N cloned and expressed in *E. coli* were purified thanks to a 6His tail (see M**ethods S1**). Then, 96-well ELISA microplates were coated with 100 ng of purified E or N proteins in carbonate buffer pH 9.6 and incubated overnight at 4°C. After blocking with 3% non-fat dry-milk in PBST (PBS + 0.1% Tween 20) during 1 hour at 37°C, plates were washed with PBST and incubated 2 hours at 37°C with serial two-fold dilutions, in PBST+1% non-fat dry-milk, of sera from 2 rabbits immunized with *P. acanthamoebae* and of corresponding pre-immune sera. After 3 subsequent washes with PBST, plates were incubated 1 hour at 37°C with horseradish peroxidase-conjugated anti-rabbit IgG (Cell Signaling, Allschwill, Switzerland) diluted 1∶1000 in PBS + 1% non-fat dry-milk. Plates were washed 5 more times with PBST. O-phenylenediamine dihydrochloride (OPD) in citrate buffer was used as substrate for the peroxydase. After 15 minutes incubation, the optical density was read at 492 nm using an ELISA reader (Multiskan Ascent, Thermo Scientific, Waltham, USA).

### Additional Methods

Descriptions of sera from immunized rabbits, immunoblot analysis, mass spectrometry and cloning, expression and purification of immunogenic proteins E and N are available in **Methods S1**.

## Supporting Information

Methods S1 Supplementary Methods
(0.03 MB DOC)

Table S1 Identification of *P. acanthamoebae* immunogenic proteins. Putative protein description of each immunogenic protein identified by mass spectrometry, according to BLASTP results against nr database or *Chlamydiales* genomes, respectively. Note that one protein spot, as for exemple F or P, can match with similar MS scores with two different ORFs, resulting in two different protein descriptions. Moreover, different spots such as D, T and U, can match with different ORFs which have the same protein description and thus correspond to orthologous or paralogous proteins.
(0.03 MB XLS)

Table S2 Western blot recognition of identified immunogenic proteins with human sera. Immunogenic proteins identified by mass spectrometry (see Table S1) were tested by western blotting of 2D gels with two sera positive for *P. acanthamoebae*, two sera positive for *C. pneumoniae*, one serum positive for *C. psittaci* and two sera negative for most *Chlamydiales*. Results are evaluated in the perspective of a *Parachlamydia* specific serological diagnostic test.
(0.03 MB XLS)

Table S3 Identification of *P. acanthamoebae* most abundantly expressed proteins. Putative protein description of each open reading frame identified by mass spectrometry (see Figure S1), according to BLASTP results against nr database or *Chlamydiales* genomes, respectively. Note that one protein spot, as for exemple 20 and 29, can match with similar MS scores with two different ORFs, resulting in two different protein descriptions. Moreover, different spots such as 35 and 36, can match with different ORFs which have the same protein description and thus correspond to orthologous or paralogous proteins.
(0.05 MB XLS)

Figure S1 2D map of most abundantly expressed *P. acanthamoebae* proteins. Proteins of *P. acanthamoebae* elementary bodies were separated by 2D gel electrophoresis and stained with Coomassie blue. Spots successfully identified by mass spectrometry are numbered A–S for immunogenic proteins and 1–29 for non immunogenic proteins (See Table S1 and Table S3).
(5.01 MB TIF)

Figure S2 Genetic organization of identified T3SS genes. The conserved genes are represented by different colors according to their respective functions. Hypothetical proteins are represented in light gray and genes encoding for proteins with identified functions likely not involved in T3SS are represented in dark gray. Capital letters refer to sct gene names according to the unified nomenclature suggested by Hueck in 1998 (Microbiol Mol Biol Rev 62: 379–433). sycE and sycD: genes encoding for SycE-like and SycD/LcrH-like T3SS chaperones. All SycD/LcrH predicted T3SS chaperones contain conserved tetratricopeptide repeats domains (TPRs).
(6.98 MB TIF)
