# Supplementary material for: High Throughput Sequencing and Proteomics to Identify Immunogenic Proteins of a New Pathogen: The Dirty Genome Approach
Source: PLoS One. 2009 Dec 23;4(12):e8423. doi: 10.1371/journal.pone.0008423 (PMC2793016; doi:10.1371/journal.pone.0008423)
Supplement: Methods S1 — Supplementary Methods (0.03 MB DOC) [file pone.0008423.s001.doc]

## METHODS S1

## Sera from immunized rabbits

Rabbits were inoculated 4 times at day 0, 14, 28 and 56 with 500 μl of PBS containing 5x10^8^ heat-inactivated *Parachlamydia*. Bleedings were obtained before immunization (pre-immune sera) and at day 90.

**Immunoblot analysis**

Nitrocellulose membranes were blocked by 2 hours incubation with 5% non-fat dry-milk in Tris-buffered saline with 0.05% Tween 20 (TBS) , washed 3 times with TBS, 0.5% milk and incubated overnight at 4°C with sera diluted in TBS, 0.5% milk. Membranes were probed either with human sera (dilution 1/64) or with sera of immunized rabbits (dilution 1/25). After 3 subsequent washes with TBS, 0.5% milk, membranes were probed with horseradish peroxidase-conjugated goat anti-human IgG (Chemicon, Temecula, CA, 1:5000), or horseradish peroxidase-conjugated goat anti-rabbit IgG (Cell Signaling, Allschwill, Switzerland, 1:1000). Finally, membranes were washed 3 times with TBS and immunoreactive spots were detected with a chemiluminescence-based kit (LiteAblot^TM^, Euroclone SpA, Pero, Italy).

**Mass spectrometry**

For identification of proteins, Coomassie Blue stained spots were excised from 2D gels and transferred to special 96-well plates (Perkin Elmer Life Sciences). In-gel proteolytic cleavage with sequencing-grade trypsin (Promega, Madison, WI, USA) was performed in the robotic workstation Investigator ProGest (Perkin Elmer Life Sciences) according to the protocol of Shevchenko et al. 2006 [1]. Digests were evaporated to dryness and resuspended in 3 μl alpha-cyano-hydroxycinnamic acid matrix (5 mg/ml in 60% (v:v) acetonitrile:water), of which 0.7 μl were deposed in duplicate on a target plate.

MALDI-MS-MS analysis was performed on a 4700 Proteomics Analyser (Applied Biosystems, Framingham, MA, USA). After MALDI-TOF MS analysis, internal calibration on trypsin autolysis peaks and subtraction of matrix peaks, the 10 most intense ion signals were selected for MS/MS analysis. Non-interpreted peptide tandem mass spectra were used for direct interrogation of *P. acanthamoebae* open reading frames, *P. amoebophila* proteins or SPTrembl databases using Mascot 2.0 (http://www.matrixscience.com). The mass tolerance for database searches was 50 ppm. With the parameters used, the threshold for statistical significance (p<0.05) corresponded to a MASCOT score of 17, but only scores greater than 30 were considered. Proteins scoring above 80 were automatically considered as valid, while all protein scoring between 30 and 80 were manually validated. Validation included examination of the peptide rms mass error of individual peptide matches. When proteins could not be identified by MALDI-TOF MS analysis, spots were reanalyzed by LC-MS followed by the same identification procedure as described above.

**Cloning, expression and purification of immunogenic proteins E and N**

The ORFs corresponding to the immunogenic proteins E (pah_c004o016) and N (pah_c008o010) were amplified by PCR and cloned into the pET28 vector (Novagen EMD Chemicals Inc, San Diego, USA). This vector system allows to express the protein of interest fused with a N-terminal 6 His tail. Protein expression was induced with 1 mM isopropyl-β-D-thiogalactopyranoside (IPTG, Qbiogen, Basel, Switzerland). The bacterial pellet was resuspended in lysis buffer (50 mM NaH_2_PO4 H_2_O, 300 mM NaCl and 10 mM imidazole, pH 8.0 (non denaturing conditions for PahE) or 100 mM NaH_2_PO4 H_2_O, 10 mM Tris, 8M urea, pH 8.0 (denaturing conditions for PahN)) containing 1 mg/ml lysozyme, 1x Halt Protease Inhibitor (Pierce Biotechnology Inc, Rockford, USA), 5 μg/ml DNAseI and 8 μg/ml RNAseA and lysed by short pulses of sonication on ice. After a high speed centrifugation to remove debris, bacterial lysate was loaded on a nickel column (HIS-Select^TM^ Spin Columns, Sigma-Aldrich, Missouri, USA) to purify the 6 His-tagged protein. The column was washed twice with washing buffer (50 mM NaH_2_PO4 H_2_O, 300 mM NaCl and 20 mM imidazole, pH 8.0 (non denaturing conditions) or 100 mM NaH_2_PO4 H_2_O, 10 mM Tris, 8M urea, pH 6.3 (denaturing conditions) and the protein of interest eluted either by adding 250 mM imidazole to the last solution (non denaturing conditions) or by decreasing the pH of the wash solution first to 5.9 and then to 4.5 (denaturing conditions). The purified protein fractions were pooled, dialysed against PBS to remove urea if necessary and the final concentration was determined using a Bradford assay (Quick Start^TM^ Bradford Protein Assay, Biorad, Hercules, USA). Purified recombinant proteins were subjected to SDS-PAGE and transferred to nitrocellulose membrane before probing with relevant rabbit or human sera or used as antigens in ELISA tests.

**REFERENCES**

1. Shevchenko A, Wilm M, Vorm O, Mann M (1996) Mass spectrometric sequencing of proteins silver-stained polyacrylamide gels. Anal Chem 68: 850-858.
