# Supplementary figures and images for: High Throughput Sequencing and Proteomics to Identify Immunogenic Proteins of a New Pathogen: The Dirty Genome Approach
Source: PLoS One. 2009 Dec 23;4(12):e8423. doi: 10.1371/journal.pone.0008423 (PMC2793016; doi:10.1371/journal.pone.0008423)

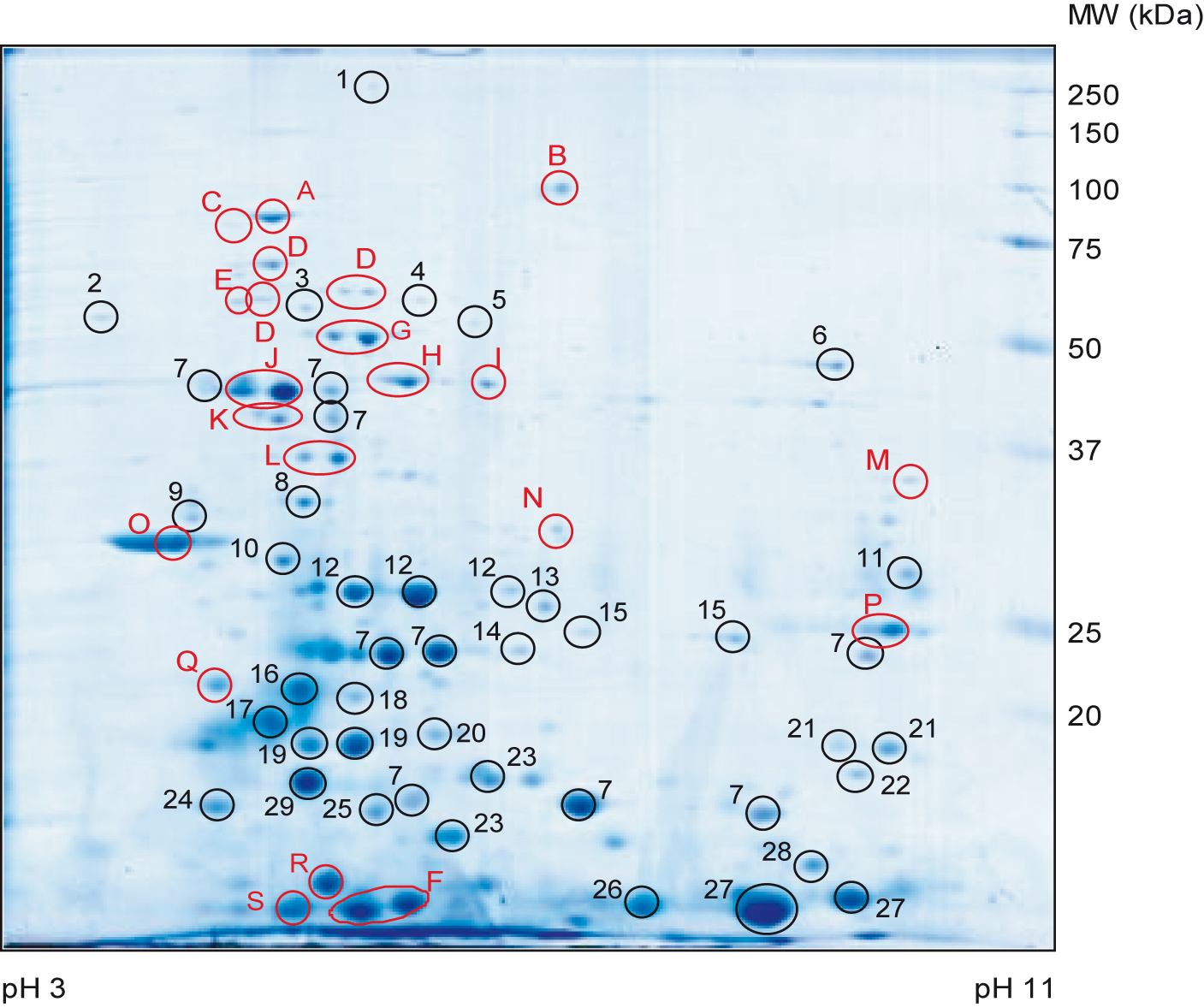

Supplement: Figure S1 — 2D map of most abundantly expressed P. acanthamoebae proteins. Proteins of P. acanthamoebae elementary bodies were separated by 2D gel electrophoresis and stained with Coomassie blue. Spots successfully identified by mass spectrometry are numbered A–S for immunogenic proteins and 1–29 for non immunogenic proteins (See Table S1 and Table S3). (5.01 MB TIF) [file pone.0008423.s005.tif]

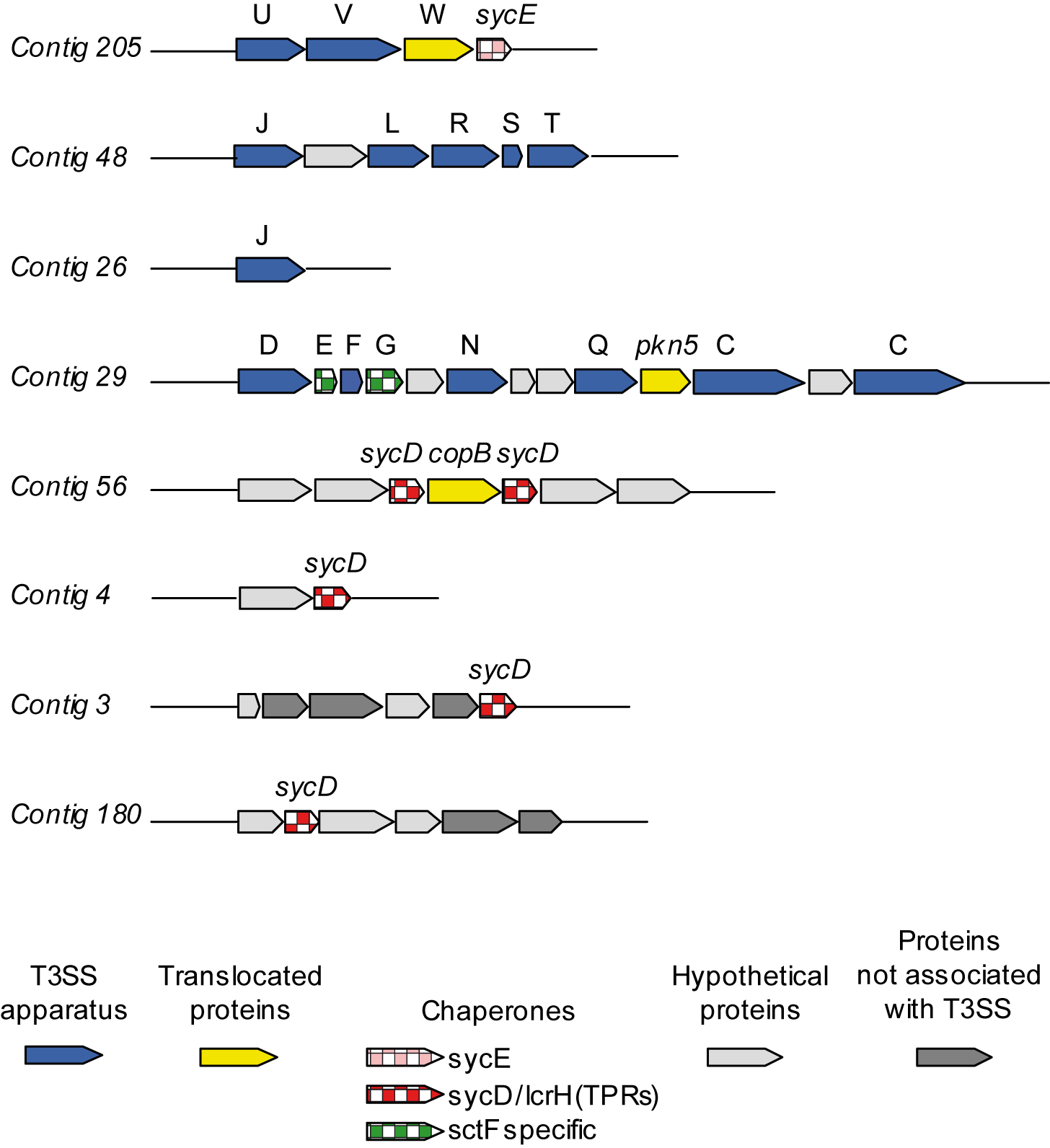

Supplement: Figure S2 — Genetic organization of identified T3SS genes. The conserved genes are represented by different colors according to their respective functions. Hypothetical proteins are represented in light gray and genes encoding for proteins with identified functions likely not involved in T3SS are represented in dark gray. Capital letters refer to sct gene names according to the unified nomenclature suggested by Hueck in 1998 (Microbiol Mol Biol Rev 62: 379–433). sycE and sycD: genes encoding for SycE-like and SycD/LcrH-like T3SS chaperones. All SycD/LcrH predicted T3SS chaperones contain conserved tetratricopeptide repeats domains (TPRs). (6.98 MB TIF) [file pone.0008423.s006.tif]
